# Genetic Variants Flanking the *FGF21* Gene Were Associated with Renal Function in Chinese Patients with Type 2 Diabetes

**DOI:** 10.1155/2019/9387358

**Published:** 2019-05-07

**Authors:** Weihui Yu, Hong Zhu, Xiong Chen, Xuejiang Gu, Xingxing Zhang, Feixia Shen, Weiping Jia, Cheng Hu

**Affiliations:** ^1^Department of Endocrinology, The First Affiliated Hospital of Wenzhou Medical University, Ouhai District, Wenzhou, 325015 Zhejiang, China; ^2^Shanghai Diabetes Institute, Shanghai Key Laboratory of Diabetes Mellitus, Shanghai Clinical Center for Diabetes, Shanghai Jiao Tong University Affiliated Sixth People's Hospital, 600 Yishan Road, Shanghai 200233, China; ^3^Institute for Metabolic Disease, Fengxian Central Hospital Affiliated to Southern Medical University, Shanghai 201499, China

## Abstract

**Aims:**

Fibroblast growth factor 21 (FGF21) is closely linked with metabolic disorders including diabetes. Evidences suggested that FGF21 may play a protective role against diabetic kidney disease (DKD). However, the relationship between genetic variants in the *FGF21* gene region and DKD remains unknown. In our study, we aimed to investigate the association of genetic variations in this gene region with DKD and DKD-related clinical traits.

**Materials and Methods:**

We recruited 1340 Han Chinese participants with type 2 diabetes, including 596 DKD patients and 744 patients who was diagnosed with diabetes for more than 5 years but did not progress to DKD. Three single-nucleotide polymorphisms were selected (rs2071699, rs838136, and rs499765) and genotyped. The association between these SNPs and DKD as well as DKD-related quantitative traits was analyzed.

**Results:**

We did not find any significant association between these SNPs and susceptibility to DKD in the present study. However, a significant association with estimated glomerular filtration rate (eGFR) was detected: in the non-DKD group, rs838136 was significantly associated with eGFR under an additive model (*β* = 0.013 ± 0.006, *P* = 0.0295, *β* was calculated for log_10_eGFR) as well as a recessive model (*P* = 0.0385) and rs499765 was associated with eGFR under a dominant model (*P* = 0.0411) and in the DKD group, rs499765 showed a trend toward association with eGFR under an additive model (*β* = −0.022 ± 0.012, *P* = 0.0820, *β* was calculated for log_10_eGFR) and showed a significant association with eGFR under a dominant model (*P* = 0.0182).

**Conclusions:**

Our findings indicated that genetic variations adjacent to *FGF21* were associated with eGFR in Chinese diabetic patients.

## 1. Introduction

Diabetes has become one of the most common chronic diseases in the world, and the prevalence of the disease has experienced an exploding increase in recent years, especially in the Chinese population [1]. As an important microvascular complication of diabetes, diabetic kidney disease (DKD) is the most frequent cause of end-stage renal disease (EASD) in different countries [2]. A genetic basis has been suggested for this disease according to the evidence of familial clustering of DKD [3] and higher risks of DKD in certain racial groups [4]. Thus, investigation of the genetic profile of DKD could reveal the underlying mechanism of the disease.

FGF21 is an endocrine member of the fibroblast growth factor (FGF) gene family, predominantly expressed in the liver. It exerts beneficial metabolic effects in animal models, such as reducing blood glucose and triglyceride levels, increasing insulin sensitivity, suppressing hepatic glucose production, increasing expenditure, and losing weight [5–8]. So it is recognized as a novel metabolic regulator, and its therapeutic potential has also been suggested for the treatment of metabolic disorders including diabetes and obesity.

Recently, studies have suggested that FGF21 may play a role in the pathogenesis of DKD. Kim et al. revealed that daily administration of FGF21 in diabetic db/db mice markedly decreased urinary albumin excretion and mesangial expansion, suppressed profibrotic molecule synthesis, and improved renal lipid metabolism and oxidative stress injury [9]. Cheng et al. found that fenofibrate prevents the development of diabetic nephropathy in mice with type 1 diabetes via upregulating FGF21 and stimulating PI3K/Akt/GSK-3*β*/Fyn-mediated activation of the Nrf2 pathway [10]. Furthermore, a recent study compared the therapeutic effect of rhFGF21 and PEG-rhFGF21 on diabetic nephropathy in diet-induced obesity (DIO) mice, and the results showed that PEG-rhFGF21 was more efficacious in ameliorating functional and morphological abnormalities induced by diabetic nephropathy in db/db and DIO mice [11]. However, whether genetic variants in the *FGF21* gene region relate to DKD is still unknown. So we aimed to evaluate the association of genetic variants in the *FGF21* gene region with DKD and its related quantitative traits in this study.

## 2. Materials and Methods

### 2.1. Subjects

1340 unrelated Han Chinese participants were recruited from the Shanghai Diabetes Institute Inpatient Database of Shanghai Jiao Tong University Affiliated Sixth People's Hospital, including 596 DKD patients (DKD group) and 744 patients who was diagnosed with diabetes for more than 5 years but did not progress to DKD (non-DKD group). All subjects were diagnosed with type 2 diabetes according to the 1999 WHO criteria (https://www.who.int/entity/diabetes/currentpublications/en). Patients with estimated glomerular filtration rate (eGFR) < 90 mL/min per 1.73 m^2^, albuminuria ≥ 30 mg/24 h, or ACR (albumin-to-creatinine ratio) ≥ 30 *μ*g/mg were diagnosed with DKD.

### 2.2. Clinical Measurements

Anthropometric and biochemical traits related to diabetes were collected in detail for each participant, including height, weight, blood pressure, fasting glucose, 2-hour glucose, glycated hemoglobin A1c (HbA_1c_), serum lipid profile, liver function, and renal function. The albuminuria level was measured using the samples from 24-hour urine collection, which was repeated in three consecutive days, and then the mean values were used for further analysis. eGFR was calculated by using a Chinese population-specific formula derived from the Modification of Diet in Renal Disease (MDRD) equation [12].

### 2.3. SNP Selection, Genotyping, and Quality Control Analysis

We chose three SNPs flanking the *FGF21* gene on chromosome 19 (all within 8 kb) including rs2071699, rs838136, and rs499765. All these SNPs were genotyped using the MassARRAY Compact Analyzer (Sequenom, San Diego, CA, USA), with matrix-assisted laser desorption ionization time-of-flight mass spectroscopy detecting primer extension of multiplex products. All SNPs met the quality control thresholds with a genotyping call rate over 90%. The minor allele frequencies for rs2071699, rs499765, and rs838136 were 28.9%, 41.6%, and 39.3%, respectively.

### 2.4. Statistical Analysis

Allele frequencies were determined by gene counting. The Hardy-Weinberg equilibrium tests were then performed for all of the SNPs in the DKD group and the non-DKD group separately. The *χ*^2^ test was conducted to compare the allele frequencies of each SNP between the two groups, and multivariable logistic regression analysis was used to examine the differences of genotype distribution between the two groups, with adjustment for confounding factors. A generalized linear model was applied to test the effects of each SNP on quantitative traits, adjusting for the covariates. eGFR and 24-hour urinary albumin excretion rate (AER) were log_10_ transformed before linear regression because of their skewed distribution. All these analyses were conducted using SAS software (version 8.0; SAS Institute, Cary, NC, USA), with a two-tailed *P* value of <0.05 considered statistically significant.

## 3. Results

**Figure 1:**
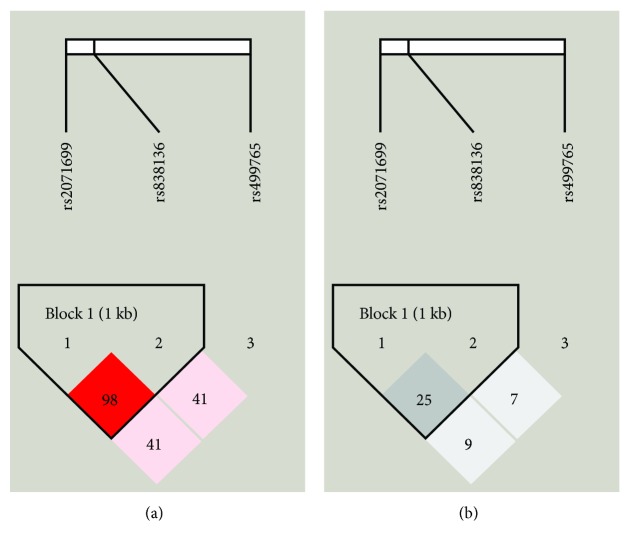
Linkage disequilibrium analyses for SNPs tested in the *FGF21* gene region. (a) Shades of pink indicate the strength of pairwise linkage disequilibrium based on |*D*′|, and numbers represent |*D*′| which is expressed as a percentage. (b) Shades of grey indicate the strength of pairwise linkage disequilibrium based on *r*^2^, and numbers represent *r*^2^ which is expressed as a percentage.

All of the SNPs conformed to the Hardy-Weinberg equilibrium (Table 1). The linkage disequilibrium pattern of these SNPs is shown in Figure 1. rs2071699 and rs838136 constructed a haplotype block (block 1) in this region with |*D*′|=0.98 and *r*^2^ = 0.25. The clinical characteristics of both groups are presented in Table 2. Significant differences were detected between the DKD group and non-DKD group with respect to gender, duration of diabetes, BMI, systolic blood pressure (SBP), diastolic blood pressure (DBP), HbA_1c_, AER, eGFR, uric acid, creatinine, blood urea nitrogen, total cholesterol, and total triglycerides (*P* < 0.05, Table 2).

We first evaluated the association between genetic variants and DKD susceptibility. As shown in Table 3, the allele frequencies of the three SNPs did not differ between the DKD group and the control group (*P* > 0.05). We also compared the genotype distribution of these SNPs between the two groups using logistic regression analysis with adjustment for age, gender, BMI, duration of diabetes, HbA_1c_, SBP, and DBP. However, no significant difference was found either (*P* > 0.05). Furthermore, there is no difference of haplotype distribution between the DKD and non-DKD groups (Table 4).

We then investigated the effects of SNPs on the quantitative traits related to DKD, including eGFR and AER. In the non-DKD group, rs838136 was significantly associated with eGFR under an additive model (*β* = 0.013 ± 0.006, *P* = 0.0295, Table 5) as well as a recessive model (*P* = 0.0385, Table 5) and rs499765 was associated with eGFR under a dominant model (*P* = 0.0411, Table 5). In the DKD group, rs499765 showed a trend toward association with eGFR under an additive model (*β* = −0.022 ± 0.012, *P* = 0.0820, Table 5) and showed a significant association with eGFR under a dominant model (*P* = 0.0182, Table 5). As for AER, no evidence of association was found for the SNPs in either group (Table 6).

## 4. Discussion

As a potent metabolic regulator, FGF21 has been closely related to obesity-related disorders, such as hyperglycemia, dyslipidemia, insulin resistance, and hepatosteatosis [5–8]. There were also supporting evidences that suggested that FGF21 may play a protective role against DKD [9–11], through both improvement of systemic metabolic alterations and antifibrotic effects [9]. We attempted to examine the association between genetic variants in the *FGF21* gene region and DKD in a Chinese population in the current study. However, we did not detect any significant association with the susceptibility to DKD for this gene region. Haplotype analysis did not show any significant result either. Since our sample size was relatively small, the association between the *FGF21* gene region and DKD should be further explored in the Chinese population with a larger sample size.

eGFR and albuminuria are the main phenotypes of DKD [13]. A few studies have suggested that serum FGF21 levels were linked with eGFR as well as albuminuria in diabetic patients. A prospective study demonstrated an independent association between elevated serum FGF21 levels and eGFR decline; thus, the circulating FGF21 level was proposed as a predictor for progressive kidney disease in subjects with type 2 diabetes and normoalbuminuria [14]. This is in accordance with the results revealed by our study that rs838136 and rs499765 were significantly associated with eGFR after adjusting for confounding factors. Individuals who carried more G alleles of rs838136 showed lower eGFR, which means that the G allele of rs838136 may be a risk factor for eGFR decline in the non-DKD group. Individuals with the GG genotype of rs499765 showed lower eGFR than those with the CC+CG genotype. rs838136 and rs499765 are both located in the flanking region of the *FGF21* gene, and they may affect the transcriptional regulation, resulting in abnormal splicing, or the translational dynamics of *FGF21*. The mechanism underlying the link between the *FGF21* gene region and eGFR is still unknown and should be further investigated by functional studies. Evidences also showed the independent association of FGF21 levels with urinary albumin excretion (UAE) or albuminuria in type 2 diabetic patients [15, 16]. However, no evidence of association with AER was found for variants in the *FGF21* gene region in our study.

Several limitations should be noted in the current study. First, the sample size is relatively small and only Southern Chinese population was included, which limits the power of our study. Secondly, we did not measure the circulating FGF21 levels in this study. Further investigations with a larger sample size as well as functional studies are needed to elucidate how FGF21 is involved in renal function in patients with type 2 diabetes.

In conclusion, our data indicated that genetic variants adjacent to *FGF21* were associated with eGFR in subjects with type 2 diabetes. Further genetic and functional studies are necessary to replicate the association and illuminate the underlying mechanisms.

## Figures and Tables

**Table 1:** Hardy-Weinberg equilibrium tests.

Group	*n*	rs2071699		rs499765		rs838136	
		CC/CT/TT	*P* value	GG/GC/CC	*P* value	AA/AG/GG	*P* value
DKD group	596	290/239/42	0.4479	201/263/104	0.2716	199/282/93	0.6791
Non-DKD group	744	357/306/58	0.5000	244/349/124	0.9668	282/320/113	0.1650

**Table 2:** Clinical characteristics of the study samples.

Parameters	Total	Non-DKD group	DKD group	*P* value
*n*	1340	744	596	
Male/female	637/703	304/440	333/263	**<0.0001**
Age (years)	64 (55, 72)	64 (56, 72)	64 (54, 73)	0.4578
Duration of diabetes (years)	10 (7, 14)	10 (8, 14)	9 (3, 14)	**<0.0001**
BMI (kg/m^2^)	24.3 (21.9, 26.7)	23.7 (21.6, 26.2)	24.9 (22.3, 27.2)	**<0.0001**
SBP (mmHg)	135 (120, 150)	130 (120, 150)	140 (130, 150)	**<0.0001**
DBP (mmHg)	80 (75, 90)	80 (70, 85)	80 (80, 90)	**<0.0001**
HbA_1c_ (%)	8.7 (7.3, 10.3)	8.3 (7.1, 10.0)	9.1 (7.5, 10.9)	**<0.0001**
AER (mg/24 h)	21.0 (8.4, 77.2)	9.2 (6.4, 14.5)	89.3 (46.3, 288.8)	**<0.0001**
eGFR (mL/min/1.73 m^2^)	116.5 (94.4, 140.7)	120.7 (102.8, 141.8)	108.9 (81.2, 136.3)	**<0.0001**
Uric acid (*μ*mol/L)	309 (250, 371)	293 (241, 351)	330 (271, 397)	**<0.0001**
Creatinine (*μ*mol/L)	68 (56, 83)	64 (54, 75)	74 (59, 96)	**<0.0001**
Blood urea nitrogen (mmol/L)	5.8 (4.9, 7.1)	5.7 (4.8, 6.8)	6.1 (4.9, 7.8)	**<0.0001**
TC (mmol/L)	4.6 (4.1, 5.4)	4.6 (4.0, 5.2)	4.7 (4.1, 5.5)	**0.0003**
TG (mmol/L)	1.4 (1.0, 2.1)	1.3 (0.9, 1.9)	1.6 (1.0, 2.3)	**<0.0001**

Data are shown as median (interquartile range) or mean ± SD. *P* value refers to the comparison of parameters between the DKD group and the non-DKD group. *P* values < 0.05 are shown in bold. DKD: diabetic kidney disease; BMI: body mass index; SBP: systolic blood pressure; DBP: diastolic blood pressure; AER: 24-hour urinary albumin excretion rate; eGFR: estimated glomerular filtration rate; TC: total cholesterol; TG: total triglycerides.

**Table 3:** Associations between SNPs and DKD susceptibility in type 2 diabetic patients.

SNP	Major/minor allele	Major allele frequency		*P*_allele_	OR (95% CI)	Genotype count		*P*_genotype_	OR (95% CI)
		DKD	Non-DKD			DKD AA/Aa/aa^†^	Non-DKD AA/Aa/aa^†^		
rs2071699	C/T	0.717	0.707	0.5845	1.049 (0.884, 1.245)	290/239/42	357/306/58	0.5511	1.062 (0.871, 1.296)
rs499765	G/C	0.585	0.584	0.9306	1.007 (0.860, 1.179)	201/263/104	244/349/124	0.7234	0.968 (0.811, 1.157)
rs838136	A/G	0.592	0.618	0.1818	0.897 (0.766, 1.052)	199/282/93	282/320/113	0.4146	0.928 (0.776, 1.110)

^†^AA = major allele homozygous; Aa = heterozygous; aa = minor allele homozygous. *P*_allele_ refers to the *P* value for the comparison of the allele frequency between the DKD group and the control group. *P*_genotype_ refers to the *P* value for the comparison of genotype distribution between the DKD group and the control group, which was adjusted for age, gender, BMI, duration of diabetes, systolic blood pressure, diastolic blood pressure, and HbA_1c_. The ORs with 95% CIs shown are for the major alleles of the SNPs.

**Table 4:** Haplotype analysis of block 1 in the study population.

	Haplotype frequency		*P* value
	DKD group	Non-DKD group	
CG	0.405	0.380	0.1986
CA	0.312	0.326	0.4413
TA	0.280	0.291	0.5386

**Table 5:** Associations between the SNPs and eGFR (mL/min/1.73 m^2^) in the study subjects.

Group	SNP	Major/minor allele	AA^†^	Aa^†^	aa^†^	Additive model		Dominant model		Recessive model	
						*β* (SE)^‡^	*P* value	*β* (SE)	*P* value	*β* (SE)	*P* value
DKD group	rs2071699	C/T	109.6 (82.3, 136.3)	106.0 (81.3, 134.0)	120.6 (86.5, 144.5)	-0.006 (0.014)	0.6515	-0.001 (0.018)	0.9337	-0.032 (0.035)	0.3517
	rs499765	G/C	105.6 (70.8, 134.7)	112.0 (85.8, 137.4)	109.9 (81.3, 135.2)	-0.022 (0.012)	0.0820	-0.044 (0.019)	**0.0182**	-0.007 (0.023)	0.7539
	rs838136	A/G	106.5 (81.5, 138.6)	109.1 (81.5, 133.1)	113.1 (89.4, 144.5)	-0.018 (0.013)	0.1758	-0.016 (0.019)	0.3937	-0.035 (0.024)	0.1534

Non-DKD group	rs2071699	C/T	120.3 (101.5, 141.5)	119.3 (102.9, 138.9)	122.5 (109.5, 147.6)	-0.006 (0.007)	0.3575	-0.009 (0.009)	0.3047	-0.004 (0.016)	0.7964
	rs499765	G/C	117.5 (102.1, 136.9)	123.4 (103.5, 148.0)	116.8 (103.5, 135.6)	-0.010 (0.006)	0.1159	-0.019 (0.009)	**0.0411**	-0.004 (0.012)	0.7560
	rs838136	A/G	121.7 (104.3, 146.4)	120.7 (102.2, 141.1)	114.9 (96.8, 136.6)	0.013 (0.006)	**0.0295**	0.014 (0.009)	0.1108	0.024 (0.012)	**0.0385**

Data are shown as geometric means (SE). *P* values were adjusted for age, gender, BMI, duration of diabetes, systolic blood pressure, diastolic blood pressure, and HbA_1c_. *P* values < 0.05 are shown in bold. ^†^AA = major allele homozygous; Aa = heterozygous; aa = minor allele homozygous. ^‡^*β* (SE) was calculated for log_10_eGFR and refers to major alleles. eGFR: estimated glomerular filtration rate.

**Table 6:** Associations between the SNPs and AER (mg/24 h) in the study subjects.

	SNP	Major/minor allele	AA^†^	Aa^†^	aa^†^	*β* (SE)^‡^	*P* value
DKD group	rs2071699	C/T	100.6 (48.3, 318.7)	82.2 (45.1, 283.4)	78.5 (37.1, 262.0)	0.030 (0.039)	0.4389
	rs499765	G/C	90.0 (52.4, 334.3)	89.1 (42.7, 289.0)	89.8 (44.6, 267.5)	0.030 (0.034)	0.3782
	rs838136	A/G	77.3 (43.0, 282.3)	103.2 (50.1, 292.6)	90.1 (46.1, 227.4)	-0.028 (0.035)	0.4327

Non-DKD group	rs2071699	C/T	9.5 (6.5, 14.7)	8.7 (6.0, 13.8)	9.4 (6.8, 13.6)	0.014 (0.014)	0.3311
	rs499765	G/C	9.7 (6.1, 14.1)	9.0 (6.4, 14.7)	8.5 (6.5, 14.1)	0.0006 (0.013)	0.9632
	rs838136	A/G	8.9 (6.2, 13.8)	8.8 (6.2, 14.9)	10.0 (6.9, 14.6)	-0.007 (0.013)	0.5667

Data are shown as geometric means (SE). *P* values were adjusted for age, gender, BMI, duration of diabetes, systolic blood pressure, diastolic blood pressure, and HbA_1c_. ^†^AA = major allele homozygous; Aa = heterozygous; aa = minor allele homozygous. ^‡^*β* (SE) was calculated for log_10_eGFR and refers to major alleles. AER: 24-hour urinary albumin excretion rate.

## Data Availability

The data used to support the findings of this study are available from the corresponding authors upon request.
